# Flexibility in the Energy Balancing Network of Photosynthesis Enables Safe Operation under Changing Environmental Conditions

**DOI:** 10.3390/plants9030301

**Published:** 2020-03-01

**Authors:** Berkley J. Walker, David M. Kramer, Nicholas Fisher, Xinyu Fu

**Affiliations:** 1 Plant Research Laboratory, Michigan State University, East Lansing, MI 48823, USA; kramerd8@msu.edu (D.M.K.); nefisher@msu.edu (N.F.); fuxinyu2@msu.edu (X.F.); 2 Department of Plant Biology, Michigan State University, East Lansing, MI 48823, USA; 3 Department of Biochemistry and Molecular Biology, Michigan State University, East Lansing, MI 48823, USA

**Keywords:** energy balancing, cyclic electron flux, malate valve, photorespiration, photosynthesis, C3 cycle

## Abstract

Given their ability to harness chemical energy from the sun and generate the organic compounds necessary for life, photosynthetic organisms have the unique capacity to act simultaneously as their own power and manufacturing plant. This dual capacity presents many unique challenges, chiefly that energy supply must be perfectly balanced with energy demand to prevent photodamage and allow for optimal growth. From this perspective, we discuss the energy balancing network using recent studies and a quantitative framework for calculating metabolic ATP and NAD(P)H demand using measured leaf gas exchange and assumptions of metabolic demand. We focus on exploring how the energy balancing network itself is structured to allow safe and flexible energy supply. We discuss when the energy balancing network appears to operate optimally and when it favors high capacity instead. We also present the hypothesis that the energy balancing network itself can adapt over longer time scales to a given metabolic demand and how metabolism itself may participate in this energy balancing.

## 1. Introduction

**Figure 1:**
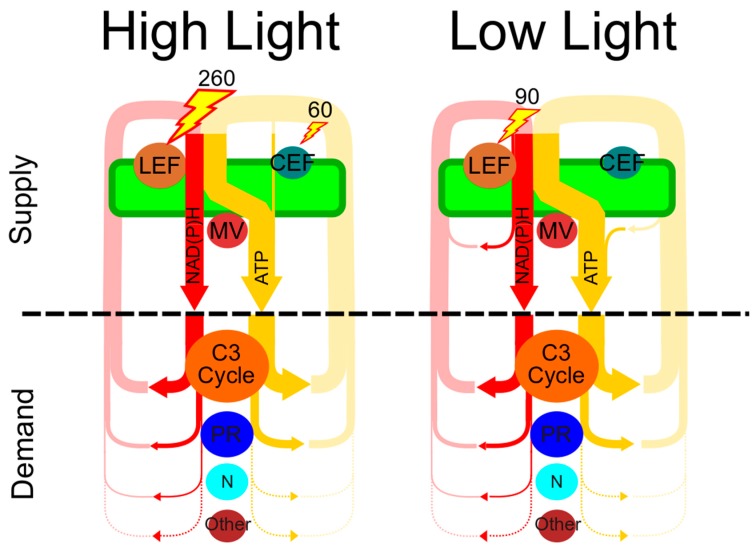
The energy balancing network configured for “high-light and low-efficiency” and “low-light and high-efficiency” conditions. Shown for each light intensity are the energy-producing supply processes of linear electron flux (LEF), cyclic electron flux around photosystem I (CEF) and the malate valve (MV), which produce reducing power (referred to generically as NAD(P)H, in red) and ATP (yellow). Metabolic demand comprises the primary ATP and NADPH consuming processes in C3 plants, the C3 cycle, photorespiration (PR), nitrate assimilation (N) and the remaining metabolic sinks for ATP and NAD(P)H (other). Numbers represent the amount of light energy absorbed by either LEF or CEF (in μmol photons m^−2^ s^−1^) needed to supply and balance the needs of metabolism. The thickness of all lines is proportional to the fluxes modeled as part of Table 1 calculated using data from Miyake et al. 2005 [1].

Photosynthetic organisms must match energy supply from the light reactions with metabolic demands to enable safe, flexible and efficient photosynthesis. Because of the interdependency between energy supply and metabolic demand, it is valuable to consider this linked energy network and not as a series of separate metabolic processes. This energy balancing network integrates ATP and reductant supply and metabolic demand to allow plants to efficiently and safely harvest energy from the sun under dynamic conditions (Figure 1). From this perspective, we will first discuss the basic mechanisms of energy balancing before presenting the demand for energy balancing under a variety of conditions. We will then discuss how the structure and efficiencies of the energy balancing network are poised to provide and turnover ATP and reducing power under a variety of conditions before exploring how the energy balancing network responds to long-term changes in metabolic demand.

## 2. Energy Balancing is Essential for Safe and Optimal Photosynthetic Systems

**Figure 2:**
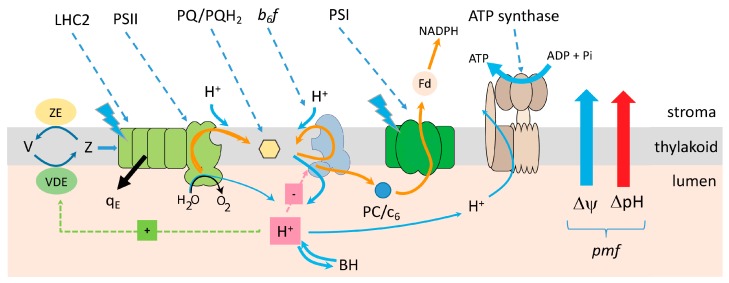
Basic Z-scheme model for the electronic and protonic circuits of the light reactions of photosynthesis, and the *pmf* paradigm for regulation of the light reactions. Scheme of linear electron flow (LEF) in oxygenic photosynthesis, in which light energy is captured by light harvesting complexes associated with photosystem II (PSII) and photosystem I (PSI), which initiates electron flow (orange arrows) from PSII, through the cytochrome *b_6_f* complex, plastocyanin (PC), to PSI and ferredoxin (Fd) and finally to NADPH. Also shown is the formation of the LEF is coupled to proton flow (blue arrows) at PSII and the cytochrome *b_6_f* complex, storing energy in the thylakoid proton motive force (*pmf*). Transfer of electrons from the lumenal to the stromal side of the thylakoid forms a transmembrane electric field (Δψ, blue arrow), while proton uptake from the stroma and deposition in the lumen lead to the formation of a transthylakoid pH gradient (ΔpH, red arrow), which together drive the synthesis of ATP from ADP + P_i_ at the thylakoid ATP synthase, storing energy in ΔGATP. The acidification of the lumen (indicated by the H^+^ in the red box) activates violaxanthin deepoxidase (VDE) which converts violaxanthin (V) to zeaxanthin (Z) and protonates the PsbS protein, which triggers the photoprotective dissipation of light energy by the q_E_ (black arrow). Lumen pH also regulates electron flow (red box with ‘-‘) to PSI by slowing the rate of PQH_2_ oxidation at the cytochrome *b_6_f* complex.

The light reactions of photosynthesis provide the chemical energy needed for plant metabolism. The core reactions of oxygenic photosynthesis involve a process called “linear electron flow” (LEF), in which light energy is used to extract electrons from water and transfer them to NADP^+^ while generating ATP from ADP and P_i_ [1,2], as detailed in Figure 2. These core processes store energy in two forms; ATP and NADPH. Extracting electrons from water and transferring them to NADP^+^, energy is stored in the two redox half reactions 4H^+^ + O_2_/H_2_O and NADP^+^ + H^+^/NADPH. In addition, the transfer of electrons results in the formation of the proton motive force (*pmf*), an electrochemical gradient of protons across the thylakoid membrane, which is dissipated by the ATP synthase to fuel the formation of ATP from ADP and P_i_. The pmf is the sum of two energetic components; an electric field component (Δ*ψ*) and the free energy stored in a chemical gradient of protons (ΔpH). Vectorial electron transfer from the lumenal to the stromal face of the thylakoid membrane, within photosystem II (PSII) and the cytochrome b_6_f complex by the Q-cycle mechanism (reviewed in [3]) and photosystem I (PSI) results in the formation of Δ*ψ*. Both Δ*ψ* and ΔpH are energetically equivalent drivers of the ATP synthase [4,5], but have very different impacts on photophysiological processes, as discussed below. One important feature of LEF is that it produces ATP and NADPH in a fixed stoichiometry, likely 2.6 ATP to 2 NADPH, or 1.28 ATP/NADPH [6].

The chloroplast must also balance the output of energy into the ATP and NADPH pools to perfectly match metabolic demands. The pool sizes of ATP and NADPH are small relative to the high fluxes of energy from the light reactions. Thus, any imbalance in the production and consumption of ATP or NADPH can rapidly lead to “metabolic congestion,” depletion or buildup of metabolic intermediates, leading to the accumulation of high energy intermediates of the light reactions within seconds [7,8,9,10]. On the other hand, if too little ATP and NADPH are produced metabolic demand is energy limited, meaning that central metabolism is sub-optimal. The “correct” output of ATP and NADPH is a moving target since metabolic demand for ATP and NADPH changes dynamically based on environmental and physiological contexts (See below and [11]). The supply of ATP and NADPH must be matched with demand both in total capacity and stoichiometrically, and therefore plants have evolved mechanisms for regulating total energy output and fine tuning ATP/NADPH production ratios.

To regulate total energy production, chloroplasts partition light energy between photochemical processes which generate ATP and NADPH (LEF) and the energy dissipating process of “non-photochemical quenching” (NPQ) [12,13,14,15,16,17]. When metabolic demand for energy is less than current supply, the major form of NPQ, termed q_E_ (for ‘energy dependent’ quenching), is triggered by acidification of the lumen (i.e., by the ΔpH component of *pmf*), through activation of violaxanthin deepoxidase, which catalyzes the conversion of violaxanthin to antheraxanthin and zeaxanthin [18], and through protonation of the antenna protein PsbS [19,20]. The ΔpH component of *pmf* also down-regulates electron flow by slowing plastoquinol (PQH_2_) oxidation by the cytochrome *b_6_f* complex, preventing accumulation of electrons on highly reducing components of PSI, a process called “photosynthetic control” (reviewed in [21,22]) and subsequent PSI photodamage. Lumen acidification is, in turn, modulated by several processes that respond to the physiological state of the cell [1]. When metabolic demand is low, the activity of the ATP synthase is also down-regulated to slow proton efflux, increasing *pmf* and down-regulation of the light reactions [1,23,24,25,26,27]. The fraction of *pmf* stored in the ΔpH or Δψ is modulated to adjust its regulatory impact of a particular *pmf* [8,26,28]. The responses of q_E_ to lumen pH may also be modulated by altering the expression of q_E_-related components [29,30,31]. Quantitatively, the dynamic range of NPQ is large, able to effectively partition from <5% to >80% of absorbed light energy to or away from energy production within tens of minutes. Importantly, even though increased light induces NPQ and decreases photochemical efficiency, the increase in total absorbed photons often more than compensates for this reduction and total LEF increases to safely produce sufficient NADPH to meet metabolic demand. Note that NPQ can only modulate total NADPH production from LEF with no change to the production stoichiometry of 1.28 ATP/NADPH.

## 3. The Structure of the Energy Network Simplifies ATP and NADPH Balancing

Once the total demand for NADPH is satisfied via the interplay between LEF and NPQ, other processes fine tune ATP/NADPH production ratios to match metabolic demand precisely. Downstream metabolism of an illuminated leaf (discussed in more detail below) requires ATP/NADPH ratios above 1.5, meaning that extra ATP is needed to achieve energy balancing. By poising metabolic demand at a higher ATP/NADPH ratio than that produced by LEF, the system can first produce the necessary NADPH, with coupled baseline production of ATP, before then producing the supplemental ATP needed for the specific metabolic context. This greatly simplifies the requirements of energy balancing since ATP and NADPH production ratios do not need to be independently re-adjusted following changes in total demand and total production capacity can be adjusted first based on a single factor (NADPH demand) before supplemental processes overcome the ATP deficit. In higher plants, three mechanisms are proposed to supply the additional ATP: (1) cyclic electron flux around PSI (CEF), (2) the malate valve and (3) the Mehler reaction. All three of these mechanisms have received extensive coverage in past reviews [6,32,33,34], and so we will focus on their basic mechanisms and relevance to the particular focus of this perspective.

## 4. Introduction to Supply-Side Mechanisms for Energy Balancing

### 4.1. Cyclic Electron Flux around Photosystem I

CEF contributes to the transthylakoid *pmf* without net production of NADPH by cycling electrons from photoexcited PSI via ferredoxin (Fd) back into the thylakoid plastoquinone (PQ) pool via the activity of Fd:PQ reductases (PQR) and the cytochrome *b_6_f* complex [35]. Aside from ATP generation, the proton gradient generated by CEF may also serve a photoprotective function by triggering q_E_ (‘energy dependent’) NPQ, although CEF in itself is not essential for this process [8,36]. Many of the details of the electron transport pathways of CEF remain obscure. At least three PQR pathways have been postulated to function in CEF, which may operate in an organism-specific manner; (i) the antimycin A-sensitive Fd:PQ reductase (FQR), which has been proposed to be associated with the PGR5 and/or PGRL1 proteins [37,38,39], (but see [8,40,41,42,43] for additional viewpoints); (ii) the respiratory Complex I-like NADPH/Fd:PQ dehydrogenase (NDH) [44,45,46,47] and iii) direct reduction of *b_6_f*-bound PQ through Q_i_-associated FNR/Fd via *b_6_* hemes *b_H_/c_i_* [41,48,49,50]. Of these CEF pathways, those utilizing the proton motive NDH complex is likely to be the most energetically efficient, with a net H^+^/2e^−^ ratio of 8 [46], with the PGR5/PGRL1 and *b_6_f* Q_i_ pathways yielding an H^+^/2e^−^ ratio of 4 by virtue of the (b_6_f-associated) Q-cycle alone [35].

The NDH pathway is, for the most part, associated with plant (and cyanobacterial) CEF, as this enzyme is absent from the majority of algal genera, although it should be noted that it is also absent from certain orchids, cacti and gymnosperms [51]. In general, the electron flux through CEF during steady-state photosynthesis in healthy, non-stressed C3 plants is considered to be small compared to LEF (i.e., ⪅15%) [8,9,50], although it is likely to be (significantly) up-regulated during environmental stress like drought or during the induction of photosynthesis in dark-adapted plants, conditions under which increased ATP demand may be expected [48,52,53]. Nevertheless, this small flux is of vital importance for balancing the ATP and NADPH demands of metabolic supply and demand. Furthermore, CEF is likely to be of particular importance to C4 photosynthetic species and aquatic algae to generate ATP and proton/ion gradients necessary for the carbon-concentrating mechanisms of these organisms [46,54,55,56]

### 4.2. The Malate Valve

The malate valve operates to adjust cellular ATP/NADPH supply by shuttling reducing power from the chloroplast to other organelles like the mitochondria via malate/oxaloacetate shuttles [32,57,58]. In the chloroplast, NADPH reduces oxaloacetate to malate via chloroplastic malate dehydrogenase (MDH). This malate is then exported from the chloroplast where it can be oxidized to form NADH in the cytosol, peroxisome or mitochondria via organelle-specific MDH enzymes. Reducing power shuttled to the mitochondria can fuel mitochondrial electron transport following transfer through the full complement of the electron transport complex proteins, generating additional *pmf* and ATP, or through only a portion of the electron transport complex proteins by dissipation of electrons via the alternative oxidase (AOX) or alternative mitochondrial electron carrier proteins. In all cases, the net effect is to increase ATP/NADPH supply either by decreasing NADPH or by simultaneously decreasing NADPH and increasing ATP. Chloroplastic NADP-MDH operates under tight light regulation via the Fd-thioredoxin (Fd-Trx) system, suggesting a role in photosynthetic energy balancing [59,60]. Importantly, the malate valve offers a way to “trade” NADPH for ATP by diverting reducing equivalence directly into mitochondrial electron transport.

### 4.3. The Mehler Peroxidase Reaction (Water–Water Cycle)

The term water–water cycle was coined by Asada (1999) [33] to indicate a process wherein electrons from LEF are extracted from water at the oxygen evolving complex of PSII, through the intermediate electron transfer chain, to PSI and to O_2_, reforming H_2_O. In plants, most of the O_2_ reduction occurs the by the Mehler peroxidase reaction (sometimes referred to as the water–water cycle (WWC)), electrons are transferred from low-potential donors (most probably F_(X/A/B_) within PSI to molecular oxygen, bypassing terminal NADP^+^ reduction, and producing superoxide. The resulting reactive oxygen species are rapidly detoxified by the activities of superoxide dismutase and the plastid ascorbate peroxidases [33]. This results in H^+^ translocation through water oxidation and the Q-cycle, without parallel NADPH production, increasing ATP/NADPH supply. Note, however, that PSI-involvement is a not a strict requirement of a WWC (i.e., the ‘traditional’ Mehler peroxidase reaction), and the activity of the plastid terminal (plastoquinol) oxidase, also serves to bypass NADPH production, albeit at low capacity [61]. Alternative forms of the WWC are also found in moss, algae and cyanobacteria, where flavodiiron proteins function as NADPH-dependent oxygen reductases [62]. While the WWC may be important under certain stress conditions, current evidence suggests that it does not operate at significant rates under a variety of conditions (i.e., <5% of LEF in tobacco when the C3 was inhibited [63]), and so it will not be further considered in the context of energy balancing [64,65].

## 5. Metabolic Demand for ATP and NADPH

While plant metabolism employs ATP and NADPH in a myriad of biochemical reactions, the vast majority of ATP and NADPH flux in an illuminated leaf enters metabolic networks at relatively few nodes of central metabolism, most notably CO_2_ assimilation and related processes, making it possible to reasonably estimate total ATP/NADPH demand [66]. Some reactions require reductive energy from alternative redox carriers (i.e., Fd or NADH) but for convenience in calculation and discussion, we will refer to reductive demand in terms of NADPH (2 e^−^) equivalents. The fixation of each CO_2_, and subsequent regeneration of the C3 cycle intermediates requires 3 ATP and 2 NADPH for a total demand of 1.5 ATP/NADPH [67]. In C3 plants growing under ambient conditions, the next largest demand for ATP and NADPH is photorespiration, which results from the molecular fixation of O_2_ by the first enzyme of the C3 cycle (rubisco, [66,68]). Photorespiration requires 3.5 ATP and 2 NADPH for complete operation, meaning that as photorespiration increases relative to CO_2_ fixation, ATP/NADPH demand increases as well.

Altering the relative rates of photorespiration and carbon fixation will alter the relative demands for ATP and NADPH. Rates of rubisco carboxylation (*V_c_*) and oxygenation (*V_o_*) determine downstream rates and energy requirements for carbon fixation and photorespiration respectively. Since *V_c_* and *V_o_* in C3 plants are constrained by rubisco kinetics, rates of each can be estimated for a given rate of net CO_2_ exchange (A) and CO_2_ and O_2_ concentration to calculate subsequent ATP and NADPH demand [68,69,70,71,72]. While these calculations have been presented in part across many publications, we compile them all herein to make their use more convenient for the non-specialist to use measured gas exchange data to calculate *V_c_*, *V_o_*, ATP and NADPH demand, and extra ATP needed above that provided from LEF (see Appendix). This quantitative framework requires several simplifying assumptions, but these estimates are close enough to show the magnitude of fluxes and relative impact between conditions.

While carbon fixation and photorespiration comprise the largest portion of central metabolic demand, other metabolic processes such as nitrate assimilation requires a significant contribution. Nitrogen assimilation in leaves involves nitrate reduction into nitrite by nitrate reductase (NR) in the cytosol, translocation of nitrite to chloroplast where it is reduced to ammonium by nitrite reductase (NiR), followed by ammonium assimilation into amino nitrogen via the glutamine synthetase (GS)-glutamine-2-oxoglutarate aminotransferase (GOGAT) pathway in the chloroplasts [73]. Nitrate assimilation to glutamine requires 5 NAD(P)H and 1 ATP. Specifically, reduction of one molecule of nitrate (oxidation state +5) to ammonium (oxidation state −3) requires eight electrons (equivalent to four NADPH), whereas the production of a glutamate via the GS-GOGAT pathway requires an additional two electrons (equivalent to 1 NADPH) and 1 ATP [66]. The reducing power required by the plastidic NiR and GS-GOGAT is supplied from photosynthetic electron transport via the reduced Fd. Higher rates of nitrate assimilation in the light than in the dark [74] reflects the tight connection between photosynthetic metabolism and nitrate assimilation. The reducing power needed for nitrate reduction via the cytosolic NR could be provided by the plastidic NAD-driven malate valve [75]. The NADPH demand for nitrate assimilation is estimated to range from ~ 0.35 to 3 μmol m^−2^ s^−1^ on an area basis, based on the nitrate assimilation rate measured by prior studies [76,77]. These rates of nitrate reduction would require ~2.5%–23% of total LEF in the sample dataset examined in Table 1, making nitrate assimilation a significant electron sink in terms of total electron flux.

Lipid biosynthesis represents another sink for NADPH and ATP consumption in plants, but quantitative estimates of its magnitude have not been reported. Lipids, being an important structural component of membranes, constitute approximately 5% to 10% of the dry weight of vegetative cells of plants [78]. The major constituents of lipids are fatty acids, which can represent up to 10% of the chemical energy of leaves on a biomass basis [79]. The synthesis and breakdown of fatty acids occur constitutively during leaf development. As much as 4% of total fatty acid content in leaves is degraded per day [80]. The turnover of fatty acids is exceeded by the rate of de novo fatty acid synthesis in non-senescent leaves. The net fatty acid accumulation generally increases during leaf expansion, with a rate ranging from 0.16 to 8 μmol carbon atoms mg^−1^ chlorophyll h^−1^ [80,81,82,83]. Plant de novo fatty acid synthesis is an energy-demanding process occurred in plastids. ATP drives the first committed step of fatty acid synthesis, the formation of malonyl-CoA from acetyl-CoA catalyzed by acetyl-CoA carboxylase. Reducing power in the form of NADPH and NADH is required for the two reductases involved in each round of fatty acid synthesis [78]. The predominant carbon source of plastidic acetyl-CoA is pyruvate, which is generated from photosynthetically fixed 3-phospho-D-glycerate (3-PGA) via the intermediate phosphoenolpyruvate. For every molecule of palmitic acid (16:0) produced, eight molecules of acetyl-CoA (generation of each acetyl-CoA from 3-PGA regenerates one ATP and one NADH), seven molecules of ATP, and 14 molecules of NAD(P)H are needed, resulting in the consumption of six molecules of NAD(P)H and surplus of one ATP collectively. Based on the total fatty acid content measured in Arabidopsis and *Brachypodium distachyon* (40 μg cm^−2^ leaf area, [83]), we estimate that the NADPH demand to maintain the 4% turnover rate of fatty acids is ~0.5 μmol m^−2^ s^−1^, which represents approximately 2% and 0.5% of the total NADPH demand under low light and high light, respectively (Table 1). Due to the small pool size of fatty acids in young leaves, the NADPH demand for fatty acid synthesis would be even smaller in the developing leaves. Although the NADPH demand for fatty acid synthesis is relatively small, this process is highly dependent on light and subject to redox regulation [84]. Nevertheless, while up to 2% of total NADPH demand has potential implications to some situations, this is insufficient to significantly affect calculations for total leaf energy balancing.

## 6. Determining the Efficiency of Energy Balancing Mechanisms

As an autotrophic organism, the energy that fuels metabolism in plants is derived ultimately from absorbed photons, providing a metric by which to gauge the efficiency of an energy balancing mechanism. Photon use efficiency has thus provided a logical objective function for approaches that assume photoautotrophs use light energy optimally (i.e., [85]), but given the massive amount of absorbed energy that is dissipated as NPQ under high light, it is not clear that light energy is always limiting to growth. Additionally, given the dynamic fluctuations in energy demand and light supply many plants face under growing conditions, it is likely that the capacity for a given energy balancing mechanism may become more important than its efficiency when light energy supply is adequate. In this section we outline the photon costs of various energy balancing mechanism and incorporate them into a quantitative framework. We then use this framework to examine past work and hypothesize that the energy balancing network operates in a high or low-efficiency mode based on light availability. To examine the energy requirements for energy balancing under various light conditions, the ATP and NADPH demand for the C3 cycle and photorespiration has been determined from past work which paired concurrent gas exchange with measurements of electron flux through PSII and PSI (Figure 1, Table 1 and [86]).

Different pathways of CEF have different costs for energy balancing, depending on how many H^+^ are pumped per electron excited by an absorbed photon. As outlined above, the highest efficiency CEF pathway proceeds through NDH, which pumps 4 H^+^/2 e^−^ (Table 2). The FQR and *b_6_f* pathways have identical yields of 2 H^+^/2 e^−^. Since 14 H^+^ are required to generate 3 ATP in the chloroplast, CEF has an ATP/photon or e^−^ stoichiometry of 0.43 via NDH and 0.21 via FQR or *b_6_f*. Additionally, the e- and photon demand for energy balancing can be calculated for the low and high-light conditions presented in Table 1 and data from Miyake et al., 2005. Under low light, 11 or 22 μmol photons m^−2^ s^−1^ are needed to produce the ATP needed to balance energy supply via NDH or FQR/*b_6_f* pathways, respectively, or between 7% and 15% of the total incident light (Table 1). Under high light, this requirement drops to 4%–8%. To gain a more complete picture of the relative energy cost of these mechanisms under their respective light conditions, the photon demand can be expressed in terms of actual absorbed photon energy that enters photochemistry by adding the rates of flux through PSII and PSI. Interestingly, this recalculation reveals that as light level increases, a greater percentage of photon energy absorbed and passed through the photosystems would need to be partitioned to CEF processes for energy balancing, specifically 11%–22% under low light and 13%–27% under high light. Under high light, however, energy from more photons is dissipated as NPQ compared to low light. If energy to NPQ is considered as excess, this means that there is more excess energy under high light that can be used to drive CEF. Specifically, based on the data from Miyake et al. 2005 [86], energy from only 48 μmol photons m^−2^ s^−1^ was dissipated via NPQ under low-light conditions, but energy from 776 μmol photons m^−2^ s^−1^ was dissipated via NPQ under high-light conditions. These numbers reveal that while photon energy could be limiting to drive CEF under low-light conditions, there appears to be enough surplus photon energy available under high light to drive CEF. This is expected because if NPQ limits excitation of PSII, it should also limit flux to PSI through LEF, but it will not necessarily limit PSI electron flow of electrons through CEF.

The energetics of the malate valve are more difficult to assess given the added complexity of transport and flexibility of mitochondrial electron transport. The initial energetics and efficiency of the malate valve are tied to LEF; eight photons produce two NADPH and 12 H^+^, resulting in 2.57 ATP. The energetics following the transport of the reducing power of 2 NADPH into mitochondrial electron transport and ATP generation depend on the e^−^/H^+^ and, more generally, the e^−^/ATP efficiency of the mitochondria. For our theoretical evaluation of malate valve energetics, we will first assume mitochondrial electron transport operates optimally and each electron contributes maximally to the proton gradient, passing through Complex I, III and IV to pump 10H^+^/2e^−^. To produce ATP, these protons pass through a ring of c-subunits of ATPase, with each full rotation producing three ATP and the number of H^+^ per rotation depending on the number of c-subunits present in the ring [87,88,89,90,91]. We assume the number of c-subunits is the same as found in animal cells since there is no available data on plant mitochondrial c-subunit number, requiring eight H^+^/3 ATP, although in yeast there are 10 c-subunits [92]. Since each molecule of ATP synthesized requires the (electroneutral) transport of one P_i_ with the associated (electrogenic) ADP^3−^/ATP^4−^ exchange activity of the mitochondrial adenine nucleotide translocase (equivalent to the uptake of an additional proton per molecule of ATP synthesized) [93,94], the final stoichiometry is 11 H^+^/3 ATP, making a theoretically maximum ATP/oxygen ratio of 2.7 [95]. This stoichiometry is closely matched in experimental measurements of the ratio of 2.6 ADP/oxygen consumed in intact mitochondria in potato [96], suggesting that these stoichiometries reasonably approximate mitochondrial respiration in plants despite the highly-branched potential of mitochondrial electron transport. Therefore, for every two NADPH (4 e^−^) that are processed via the malate valve, 5.45 ATP are produced in the mitochondria. The above discussion focuses specifically on the ATP produced via mitochondrial respiration fueled by electrons provided from the light reactions, we recognize that some ATP may be produced in the light from pyruvate produced during “dark-type” glycolysis. Exact rates of glycolysis-supplied mitochondrial respiration in the light are not available, but estimates from CO_2_ gas exchange indicate these rates are rather small compared to net assimilation and lower than rates measured in the dark [97,98,99], suggesting that the bulk of ATP generated in the mitochondria may come from other sources (such as the Mehler valve), but more information is needed to explore this in more detail.

To integrate the production stoichiometries into a complete malate valve cycle, the costs of transporting ATP from the mitochondria back into the chloroplast where it is primarily needed for the phosphorylation of C3 and photorespiratory cycle intermediates must also be considered. Transport of ATP from the mitochondria proceeds via the ADP/ATP translocase [100,101] and into the chloroplast via the plastidic ADP/ATP transporter [102,103].

The above energetics determine that the malate valve is a highly efficient ATP producer on a photon basis. For every eight photons of light energy, 2.57 ATP are produced in the chloroplast and 5.45 are produced in the mitochondria for a total ATP/photon ratio of 1, much higher than the 0.21–0.43 determined for CEF (Table 2). This high ATP/photon ratio means that much less absorbed light energy is needed for energy balancing assuming low and high-light conditions (Table 1). Specifically, only 4.7 and 18.5 μmol photons m^−2^ s^−1^ were needed under low and high light, respectively (Table 1). This comprises only 3.2$ and 1.7% of incident irradiance for the low and high light intensities.

## 7. With an Efficient Malate Valve, Why is CEF Important?

Given the theoretically greater efficiency of the malate valve, how can we explain the commonly observed participation of CEF in energy balancing? We propose that the real energy cost of CEF will depend on the light intensity and other factors. At low light, when energy capture is strongly limited by the number of photons captured by the photosystems, activating CEF will require diverting energy from LEF, limiting the overall efficiency of energy capture. However, as light input nears saturation imposed by downstream reactions, PSII efficiency drops substantially, either by decreased efficiency of antenna (increased NPQ), or by increases in the fraction of closed PSII centers. In this case, activating CEF may have little effect on the efficiency of LEF (because it is already light saturated) but will increase total energy capture, albeit with a higher fraction stored in ATP/NADPH. Indeed, experimentally, CEF appears to play a larger role in energy balancing under high, but not low irradiances when there is limiting energy available from absorbed photons [72,86].

**Figure 3:**
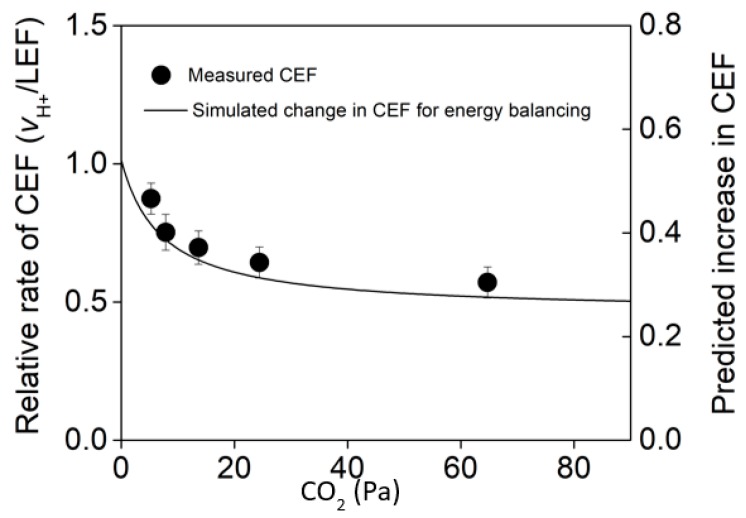
Comparison of the measured relative rate of cyclic electron flux (CEF: circle symbols) to the predicted change in CEF required to match ATP/NADPH supply with demand across CO_2_ concentration (line). Shown are n = 3–4 ± SE. This data is a replotted subset of measurements from Walker et al. (2014) [72].

For example, when high light intensities drive light-saturated photosynthetic rates, CEF shifts proportionally in response to changing CO_2_ to cover ATP deficient predicted from gas exchange [72]. These measurements were made across CO_2_ concentrations and reveal the capacity for CEF to respond to changes in leaf energy demand (Figure 3). We refer to the measured change of CEF in response to changing energy balancing requirements as the “dynamic range of CEF”. By comparing the measured dynamic range of CEF to the change predicted from gas exchange modeling, the degree by which CEF participates in energy balancing can be determined. The dynamic range of CEF can run into a self-regulating upper limit when the high rates of CEF increase the ΔpH and initiate q_E_-dependent NPQ, as occurs in shade leaves exposed to increasing light [104]. This could serve as a protective mechanism, when CEF increases ΔpH to sufficient levels, light harvesting is down-regulated and further energy mismatch is avoided when the capacity for energy balancing by CEF is reached. Interestingly, the dynamic range of CEF was minimal in response to changing energy demanding conditions when measured under low light, indicating that processes other than CEF (e.g., the malate valve) accomplish energy balancing under these low flux conditions [72].

A role for “low-light, high-efficiency” and “high-light, low-efficiency” energy balancing networks is further supported by flux balance analysis of photosynthetic systems and in mutant lines deficient in CEF. Flux balance analysis of photosynthetic systems that are optimized for energy production per photon of absorbed light predict the malate valve to be the optimal mechanism of energy balancing unless the additional costs of enzymatic interconversions are introduced into the model [105]. This is also supported in work using a modified flux balance analysis approach, which weights flux solutions based on pathway complexity [106]. As light intensity increases, absorbed light energy is actively released as NPQ, indicating that under high light, the system is no longer light limited and the energy balancing network could trade the more light-optimal malate valve for CEF. This position is supported by work showing that CEF is critical for plant growth under high, but not low-light conditions [107].

It is not clear what the advantages of CEF might be over the malate valve, but the speed and flexibility of CEF may provide an explanation. The operation of the complete malate valve requires tight coordination of enzymatic and transport activity between the chloroplast and mitochondria, limiting the dynamic range of its energy balancing capacity over short time scales. The malate valve also requires the careful coordination of two electron transport chains in separate organelles, further complicating the upregulation of this pathway under greater energy balancing demand. CEF occurs only in the chloroplast, simplifying the signaling network required to up- or down-regulate ATP production. By contrast, CEF is likely regulated by stromal ATP levels as well as stromal redox state [108] and thus may also be more rapidly responsive to alterations in energy demands, e.g., during induction or rapid changes in light, CO_2_, etc. whereas the malate valve appears to require (potentially slower) redox activation (see below). Thus, having at two routes of ATP/NADPH balancing, provides photosynthetic systems with greater flexibility to balance diverse metabolic imbalances as well as providing optimal efficiency under low light (malate valve) or more rapid/responsive responses (CEF) pathways. We further hypothesize that the baseline requirements for energy balance are achieved by the more light-optimal malate valve. This baseline activity satisfies the needs for energy balancing until greater capacity is needed, such as occurs under higher light regimes. Under high light, CEF acts as a highly flexible stop-gap to allow energy balancing to occur under dynamic conditions.

## 8. Demand-Side Energy Balancing Processes

While there is much focus on how supply-side reactions mediate energy balancing, there is less focus on how metabolic demand itself changes. For example, under increased ATP/NADPH demand, metabolism could either increase the supply of ATP via supply-side mechanisms like CEF or the malate valve or reprogram metabolism itself to use the supply more optimally. A simple example of this is in the redox regulation of the C3 cycle, where multiple redox post-translational modifications may tune activity to available reducing power availability [109].

There is additional evidence for this demand-side reprogramming to achieve energy balancing in the unique link between nitrate assimilation and photorespiration. C3 plants have lower nitrate assimilation rates when photorespiration is reduced through altered atmospheres [76,77,110,111,112]. This link could be explained if under ambient conditions, the increased demand for ATP/NADPH imposed by photorespiration is offset by increased rates of nitrate assimilation, which has a much lower ATP/NADPH demand. This would achieve ATP/NADPH balance not exclusively by increased ATP supply, but by repartitioning demand-side processes themselves. Interestingly, expression of nitrate assimilation genes increase in NADP-MDH mutants, suggesting that nitrate assimilation could be a compensatory response to achieve energy balancing when the malate valve is disrupted [75]. NADP-MDH mutants also show improved growth on nitrate-rich media [75,113]. These experiments demonstrate that nitrate assimilation and the malate valve may cooperate to maintain a baseline level of energy balancing, increasing the complexity of the energy balancing network.

## 9. The Acclimation of Energy Balancing Networks to Long-Term Change in Energy Demand

As mentioned above, plants must cope in the long term to changing ATP and NADPH demand to achieve energy balance. It is unclear whether the same mechanisms balance energy mismatches under long time scales as occur under shorter time scales. Furthermore, it is unknown to what degree energy balancing networks poise to a given demand and how this poise acclimates to changing demands. The acclimation of the energy balancing network can be investigated experimentally either via transition experiments or by examining mutants with an altered network capacity that forces flux through alternative facets of the network. We will first discuss the potential for acclimation of supply-side processes to changing energy demand before outlining how metabolic demand itself may acclimate to changes in energy balancing requirements.

According to our model of the two-component supply-side energy balancing system, malate valve activity should scale with excess ATP demand over long-term transitions to optimally use absorbed light. The largest driver of excess ATP demand for any condition is increased light, and so this model predicts that malate valve activity should increase with light. Indeed there appears to be a light and dark malate valve cycle, with the dark cycle relying on plastidic NAD-MDH and the light shuttle using plastidic NADP-MDH [58]. The switch to the NADP-MDH cycle is mediated through the light-dependent Fd-Trx system [114,115]. This activation (at least for NADP-MDH in isolated Pea chloroplasts) occurs within 10–20 min, and so activation of this component is likely integrated in our short-term measurements [116]. Activation occurs even more rapidly during a high-light transition [117]. This is an effective regulatory strategy at short time scales, since it activates malate valve activity when there is a surplus of NADPH, as when the C3 cycle is not consuming it fast enough and reduces it when there is too much NADP^+^ [58]. It is also likely that as the malate valve is reaching full capacity, CEF plays a role in vivo during very short time scales, at least in C3 plants [118].

Other factors increase malate valve capacity over longer time scales. For example, after transfer to sustained high light, NADP-MDH expression and protein levels increase, suggesting increased capacity of the malate valve following hours of exposure to the new condition [119]. Interestingly, the same response is not observed when photoperiod increases, suggesting that a photosynthetic steady-state solution must be found and that the effect is not cumulative.

Interestingly, chloroplastic *nadh-mdh* mutants show no phenotype, even under stress conditions, potentially due to additional compensatory redox strategies [75,113]. It is important to note that the malate valve shuttles reducing power not only between the chloroplast and mitochondria, but also the peroxisome during photorespiration [120]. In contrast to chloroplastic *nadh-mdh* mutants, mitochondrial *nadh-mdh* mutants lacking both MDH isoforms (*mmdh1mmdh2*) show lowered photosynthetic rates and growth rates [121]. These decreases were likely due to impaired shuttling of reducing power from the mitochondrion to the peroxisome via the malate valve to provide the reducing power for hydroxypyruvate reduction in photorespiration, a viewpoint supported using ^13^C flux analysis of *mmdh1* [122]. Indeed *mmdh1mmdh2* show reduced photorespiratory capacity, but the reduced growth and photosynthesis is not explained strictly by decreased availability of reductant to photorespiration since mutants lacking the peroxisomal MDH isoforms show an even more subtle phenotype than *mmdh1mmdh2* [123,124]. This work with mitochondrial MDH indicates that the malate valve is not strictly required for energy balancing, but it is important for optimal photosynthesis and long-term growth.

The capacity for CEF itself may also increase over longer time scales to allow for increased energy balancing demand. Notably, NDH and FQR content change under different growth conditions [51]. The ratio of PSI and PSII re-proportions when plants are grown under light regimes with outputs that favor PSI or PSII. After hours or days, this results in changes to the actual stoichiometry of PSI and PSII photosystems, in green algae [125,126,127] and plants [128]. This re-proportioning also occurs days following transition between different light qualities, which can increase the capacity for CEF [129]. At short time scales, repartitioning of light energy between PSI and PSII occurs when reduced PQ builds up and triggers the phosphorylation of the PSII light harvesting complex. These then migrate to PSI to balance out energy capture [130]. While these state transitions occur in response to long-term differences in energy supply (changes in light regimes), it is not clear if this happens in response to long-term changes in energy demand. Such a change would predict that as conditions decrease in the ratio of photorespiration, there should be a decrease in demand for CEF and, therefore, a decrease in the PSI/PSII ratio.

Measurements of PSI/PSII from plants grown under conditions of different ATP/NADPH demand did not indicate that the capacity for CEF change with energy demand via changes in photosystem stoichiometry. Specifically, there was no difference in PSI/PSII in aspen trees exposed to elevated CO_2_ (560 PPM) over a single season following 5 years of elevated high CO_2_ treatment [131]. However, this increase in CO_2_ is not expected to change the demand for CEF by all this much (~1% of LEF change). Additionally, micro-array work in soybeans exposed to 550 PPM also show no difference in photosystem expression, but interestingly show an increased expression of a mitochondrial ATP/ADP antiporter [132]. Overall, these findings do not point clearly to the acclimation of the capacity of CEF in response to changing energy demand, but the treatments resulted in relatively modest changes in energy demand and CEF was not evaluated specifically. There is clearly room for more work examining this question specifically.

## 10. Conclusions

The energy-balancing network comprises a flexible set of possibilities that enable partitioning through pathways with different ATP and NADPH production stoichiometries that require different amounts of light energy. We hypothesize that the network partitions flux through high-efficiency pathways (e.g., the Malate valve) when light is limiting and high-efficiency pathways (e.g., CEF) when light is abundant. Furthermore, the energy balancing network may adapt to long-term energy demand through enzyme expression.

## Figures and Tables

**Table 1:** Requirements for energy production for the supply and demand of the energy balancing network under low and high light in *Nicotiana tabacum*. For metabolic demand, shown are rates of CO_2_ assimilation (A), intercellular and chloroplastic CO_2_ concentration, rates of rubisco carboxylation (*v_c_*) and oxygenation (*v_o_*), rates of nitrate reduction (*V_n_*), rates of lipid production (V_l_) and total ATP and NADPH demand. For energy supply shown are photosynthetically active radiation (PAR), measured rates of electron transport through PSII (LEF) and PSI (J_PSI_), rates of linear electron flux needed to provide sufficient NADPH for metabolic demand (LEF_pred_), ATP produced from LEF_pred_ (ATP_LEF_) and the ATP deficit. For energy balancing, shown are the electron and photon demands for the ATP deficit to be provided by CEF via the NDH, FQR or *b6f* pathways or the malate valve. Details for these calculations found in the text. Values taken from Miyake et al. 2005 [1] indicated with a star (*), with remaining values calculated or assumed herein. For these calculations R_l_, Γ* and g_m_ were assumed to be 1.5 μmol CO_2_ m^−2^ s^−1^, 4.7 Pa and 6 μmol CO_2_ Pa CO_2_^−1^ m^−2^ s^−1^.

	High Light (1100 PAR)	Low Light (150 PAR)
Metabolic demand		
A (μmol CO_2_ m^−2^ s^−1^)	21.3 *	5.7 *
Intercellular CO_2_ (Pa)	23.0 *	25.0 *
Chloroplastic CO_2_ (Pa)	19.5	24.1
*v_c_* (μmol CO_2_ m^−2^ s^−1^)	30.1	8.9
*v_o_* (μmol O_2_ m^−2^ s^−1^)	14.5	3.5
*v_n_* (μmol N m^−2^ s^−1^)	1.5	0.5
*v_l_* (μmol N m^−2^ s^−1^)	0.3	0.1
Total ATP demand (μmol ATP m^−2^ s^−1^)	143	40
Total NADPH demand (μmol NADPH m^−2^ s^−1^)	97	27
Total ATP/NADPH ratio	1.47	1.45
Energy supply		
LEF (μmol m^−2^ s^−1^)	132.0 *	45.6 *
J_PSI_ (μmol m^−2^ s^−1^)	192.0 *	56.4 *
LEF_pred_ (μmol m^−2^ s^−1^)	193.9	54.4
ATP_LEF_ (μmol m^−2^ s^−1^)	124.1	34.8
ATP deficit (μmol m^−2^ s^−1^)	18.6	4.7
Energy balancing requirements via CEF		
NDH e^−^ (μmol m^−2^ s^−1^)	43.3	11.1
NDH photons (μmol m^−2^ s^−1^)	43.3	11.1
FQR/*b_6_f* e^−^ (μmol m^−2^ s^−1^)	86.6	22.2
FQR/*b_6_f* photons (μmol m^−2^ s^−1^)	86.6	22.2
Energy balancing requirements via malate valve		
e^−^ (μmol m^−2^ s^−1^)	9.3	2.4
Photons (μmol m^−2^ s^−1^)	18.5	4.7

**Table 2:** Energy requirements and efficiencies of CEF pathways and the malate valve to produce supplemental ATP. Shown are the number of absorbed photons used for the calculation of each pathway. Further details and assumptions for calculations are found in the text.

	CEF Pathways			
	NDH	FQR	*b_6_f*	Malate Valve
**Chloroplast**				
e-/photons	1	1	1	0.5
H^+^/e^−^	2	1	1	3
ATP/H^+^	0.21	0.21	0.21	0.21
ATP/photon in chloroplast	0.43	0.21	0.21	0.32
**Mitochondria**				
H^+^/e^−^	-	-	-	5
ATP/H^+^	-	-	-	0.27
ATP/photon in mitochondria	-	-	-	0.68
**Total ATP/photon**	0.43	0.21	0.21	1.00

## Abbreviations

**Abbreviation**	**Full Name**
ΔpH	The gradient potential component of the proton motive force
Δ*ψ*	Electric force component of the proton motive force
A	Net CO_2_ assimilation
AOX	Alternative oxidase
CEF	Cyclic electron flux around photosystem I
Fd	Ferredoxin
Fd-Trx	Ferredoxin-thioredoxin system
FQR	Ferredoxin:plastoquinone reductase
GS	glutamine synthetase
GOGAT	glutamine-2-oxoglutarate aminotransferase
LEF	Linear electron flow
NDH	NADPH/Ferredoxin:plastoquinone dehydrogenase (NDH)
*pmf*	Proton motive force
MDH	Malate dehydrogenase
NiR	Nitrite reductase
NPQ	Non-photochemical quenching
NR	Nitrate reductase
PQ	Plastoquinone
PQH_2_	Plastoquinol
PQR	Ferredoxin:plastoquinone reductases
PSI	Photosystem I
PSII	Photosystem II
q_E_	Energy-dependent quenching
R_l_	Respiration in the light
*V_c_*	Rate of rubisco carboxylation
*V_o_*	Rate of rubisco oxygenation

## Appendix A

### Calculating ATP and NADPH Ratios from Gas Exchange Data

While the theory behind calculating ATP and NADPH demand ratios for rubisco carboxylation (*V_c_*) and oxygenation (*V_o_*) from gas exchange data has been published on previously [6,72], the underlying derivation and final equations have been admittedly lacking. Here, we present a complete derivation, complete with underlying assumptions, appropriate to allow the non-specialist to apply these calculations to their gas exchange data. We will not attempt a full derivation of the underlying biochemical model of leaf photosynthesis, but will instead refer to the relevant equations directly as presented completely previously [133,134].

The cornerstone equation for modelling net CO_2_ assimilation (A) is the mass balance subtracting from rates of carbon fixation via *V_c_* and rates of CO_2_ loss from photorespiration and respiration in the light (R_l_). Rates of photorespiratory CO_2_ loss are calculated by multiplying CO_2_ loss per rubisco oxygenation (usually assumed to be 0.5) by *V_o_* and total A is represented by Equation (2.1) in von Caemmerer 2000 [133]:

(A1) $$A=V_{c}-0.5V_{o}-R_{l}$$

Since our goal is to use measured rates of A to estimate *V_c_* and *V_o_*, and subsequent ATP and NADPH demand, it becomes convenient to express Equation (A1) in terms of one unknown variable (*V_c_*) and then solve for *V_o_*. Equation (A1) is expressed in terms of *V_c_* in principle by combining rubisco specificity for CO_2_ relative to O_2_ (S_c/o_) with measured gas concentrations to determine what catalytic rates of *V_c_* and *V_o_* would produce the measured A. This is accomplished based on the following relationships (Equations (2.16) and (2.18) from von Caemmerer 2000 [133])

(A2) $$\phi =\frac{V_{o}}{V_{c}}$$

(A3) $$\phi =\frac{2\Gamma^{*}}{C_{c}}$$

where C_c_ is the partial pressure of CO_2_ at the site of rubisco catalysis and Γ* is the CO_2_ compensation point in the absence of R_l_ defined as

(A4) $$\Gamma^{*}=\frac{0.5O}{S_{c/o}}$$

where O is the oxygenation partial pressure. Note that since O is part of the definition of Γ*, it must be scaled according to the measurement concentration if an altered oxygen background is used during the experiment. In using Equation (A1), R_l_ is assumed or independently measured under the experimental conditions using a variety of gas exchange approaches and treated as a constant [98,135,136]. With Equations (A1)–(A3) we are able to represent the relationship between A and *V_c_* with no other unknown variables

(A5) Vc=A+Rl1−Γ∗Cc

The solution for *V_c_* can then be used with Equation (A1) to solve for *V_o_*.

(A6) $$V_{o}=\frac{V_{c}-A-R_{l}}{0.5}$$

With *V_c_* and *V_o_* determined from the above, the rate of demand for ATP (V_ATP_) and NAD(P)H (V_NADPH_) can then be determined based on the requirements for the C3 cycle (3 ATP and 2 NADPH) and photorespiration (3.5 ATP and 2 NAD(P)H, [66,67]) according to

(A7) $$V_{ATP}=3V_{c}+3.5V_{o}$$

And

(A8) $$V_{NADPH}=2V_{c}+2V_{o}$$

Of course additional energy demanding processes can be added to Equations (A6) and (A7) to determine total leaf energy demand [72], but we have limited these calculations to those most directly measured using gas exchange.

It should be noted that several of the constants assumed above require additional interpretation depending on the species and conditions they are measured under. These calculations depend on C_c_ to account for the chloroplastic supply of CO_2_, but standard gas exchange measurements can only practically resolve the concentration of CO_2_ in the intercellular airspace (C_i_). C_i_ can be converted to C_c_ assuming a simple linear conductance using Fick’s law as

(A9) $$C_{c}=C_{i}-\frac{A}{g_{m}}$$

where g_m_ is the mesophyll conductance to CO_2_ diffusion. Selecting an appropriate g_m_ to use experimentally is complicated since it varies by species, temperature and the underlying theory used for it estimation [137,138,139,140,141,142,143,144,145]. Fortunately, under most conditions, V_ATP_ and V_NADPH_ are not extremely sensitive to small errors in g_m_ assumptions, but a sensitivity analysis can be performed to confirm that the findings of a study are robust. Note that stomatal conductance has a similar impact on changing C_i_ for a given photosynthetic rate. Since stomata close during drought, this means that the ratio *V_o_/V_c_* increases under these water-limiting conditions, increasing metabolic demand for ATP/NADPH. Additionally, the temperature response of Γ* should be accounted for in addition to its linear dependence on O [138].
